# Increased Snap Counts Were Not Seen Prior to Lisfranc Injuries in the National Football League

**DOI:** 10.7759/cureus.32736

**Published:** 2022-12-20

**Authors:** Varag Abed, Rachel Fine, Rebecca Fine, Caitlin Conley, Cale Jacobs, Austin V Stone

**Affiliations:** 1 Orthopaedic Surgery and Sports Medicine, University of Kentucky, Lexington, USA

**Keywords:** national football league, nfl, injury risk, snap count, lisfranc fracture

## Abstract

Introduction

Athletes in the National Football League (NFL) subject their bodies to a great deal of physical strain, which places them at an increased risk for injury. The purpose of this study was to determine if there was an increase in snap counts played during a player’s injury game or season that may have contributed to an increased risk of sustaining a Lisfranc injury in the NFL. We hypothesized that players who play more snaps than they have in seasons prior will be at an increased risk of injury.

Methods

NFL players with Lisfranc injuries were identified by cross-referencing multiple online resources. Information on a player’s position, draft year, draft round selection, height, weight, snap counts, approximate value (AV), quarter of injury (first, second, third, fourth), quarter of a season (games one through four, five through eight, nine through twelve, thirteen through sixteen), and injured foot laterality was collected. A control group of players without a Lisfranc injury was then selected to compare performance data with our injured cohort that returned to play.

Results

Twenty-one NFL players, who met inclusion criteria, sustained a Lisfranc injury between the years 2013 and 2021. Players played significantly fewer snaps before sustaining a Lisfranc injury compared to their season average (33.9 ± 21.9 vs. 50.3 ± 15.8; p=<0.001), but when comparing the number of snaps played per game in their injury year with the number of snaps played per game over their career before injury, there was no significant difference (50.3 ± 15.8 vs. 45.7 ± 17.1; p=0.20). Most injuries occurred in either the first (42.9%) or second (33.3%) quarter of a regular season (games one through eight). During a game, the timing of most injuries was either the second (38.1%) or fourth (33.3%) quarter. There was no significant difference between injured players and controls post-injury between the number of average seasons played, AV, and snaps played per game.

Conclusion

Increased snap counts were not seen prior to Lisfranc injuries in the NFL.

## Introduction

Athletes in the National Football League (NFL) subject their bodies to a great deal of physical strain, which places them at an increased risk for injury [1]. Specifically, foot injuries account for greater than 15% of all athletic injuries, with midfoot sprains accounting for four percent in collegiate football players each year [2]. A Lisfranc injury can occur to either the ligament or bone, which causes instability when attempting to perform strength and balance maneuvers [2]. As a result, this can prove a great hindrance to a professional athlete’s ability to perform high-level movements. In the general population, this injury has a low incidence of one per 55,000 people but is becoming increasingly diagnosed in football players [2].

Recovering and returning to sport from a Lisfranc injury can take several months [3]. For NFL players, this can result in missing both games during the season in which they got injured, as well as the following ones. Although previous studies have reported on the return to play rate of athletes following Lisfranc injuries [3,4], there is limited literature on whether workload placed on athletes contributes to their injury.

The purpose of this study was to determine whether an increase in snap counts played during a player’s injury game or season may have contributed to an increased risk of sustaining a Lisfranc injury in the NFL. We hypothesized that players who play more snaps than they have in seasons prior will be at an increased risk of injury.

## Materials and methods

**Figure 1:**
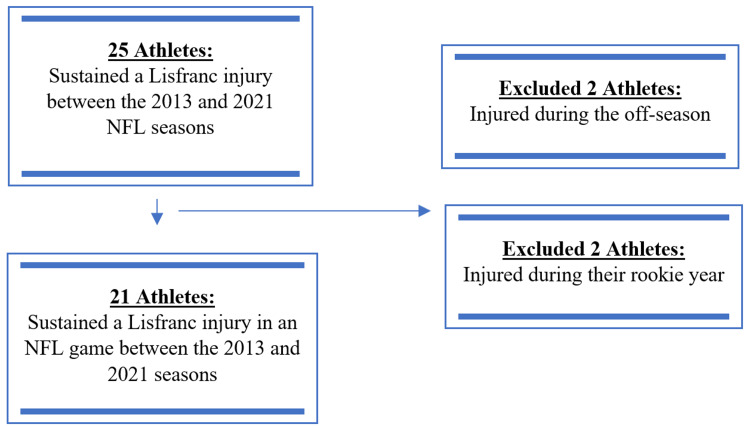
Flow Diagram Demonstrating Final Cohort Selection NFL, National Football League

NFL players with a Lisfranc injury were identified by cross-referencing multiple online resources and articles including official injury reports, press releases, game summaries, and online publications [5-9]. This method has been utilized in previous studies [10-13]. Each documented case of a Lisfranc injury was verified by a minimum of two separate sources. Inclusion criteria included players who sustained a Lisfranc injury in an NFL game between the 2013 and 2021 seasons and played in at least one season prior to injury. Exclusion criteria included players who were injured in the off-season or during their rookie season. Twenty-one NFL players met inclusion criteria for this study (Figure 1).

A customized Excel spreadsheet was created to encompass all the data extracted from the online resources. Information on a player’s draft year, round selection, height, and weight was found using the Pro Football Reference website [14]. Players were classified into the following positions: quarterback, running back, wide receiver, tight end, offensive guard, center, offensive tackle, defensive end, defensive tackle, linebacker, and cornerback. These positions were grouped accordingly for analysis: quarterback, running back, receiver (wide receiver, tight end), offensive lineman (offensive guard, center, offensive tackle), defensive lineman (defensive end, defensive tackle), linebacker, and cornerback.

The Pro Football Reference database was also used to collect data points into a customized Excel spreadsheet which included snap counts and AV [14]. The AV has been utilized in the NFL to create a standard metric to compare the performance of players of different positions [15]. Pro Football Reference began recording snap counts in the 2012 NFL season [14]. Date of injuries, quarter of injury (first, second, third, fourth), quarter of a season (games one through four, five through eight, nine through twelve, thirteen through sixteen), and injured foot laterality were also collected from the online resources cited above. A control group of players without a Lisfranc injury was selected to compare performance data with our injured cohort that returned to play. Injured players were matched to a control group based on the position, average age, body mass index (BMI), seasons played before injury, AV one year before injury, and snaps played per game during the injury season. The injury season was considered the “index year” similar to previous studies [16,17]. Previous studies reporting a Lisfranc injury in athletes have had similar or lower sample sizes [3,4,18-20].

Statistical analysis

Descriptive statistics were tabulated for all measured outcomes. Categorical variables were summarized using the frequency and percentage. Continuous outcome variables were summarized using the mean and standard deviation, unless notable skewness was observed via plots and Shapiro-Wilk testing, in which case the median and interquartile range (IQR) were used.

Due to notable skewness, differences in games played were analyzed using Wilcoxon rank-sum and signed-rank tests, as appropriate. Differences in all other quantitative outcomes were analyzed using paired and unpaired Welch two-sample t-tests, as appropriate. Across all analyses, a p-value of less than 0.05 was considered significant. All analyses were completed in R 4.2.1 (R Foundation for Statistical Computing; Vienna, Austria). 

## Results

Twenty-one NFL players, who met inclusion criteria, sustained a Lisfranc injury between the years 2013 and 2021. Of the 21 players, 18 underwent surgical correction. On average, NFL players played significantly fewer snaps before sustaining a Lisfranc injury compared to their season average (33.9 ± 21.9 vs. 50.3 ± 15.8; p=<0.001), but when comparing the number of snaps played per game in the index year with the number of snaps played per game over their career before the index year, there was no significant difference (50.3 ± 15.8 vs. 45.7 ± 17.1; p=0.20). Most injuries occurred in either the first (42.9%) or second (33.3%) quarter of the regular season (games one through eight). During a game, the timing of most injuries was either the second (38.1%) or fourth (33.3%) quarter (Table 1).

**Table 1:** Player Demographics (n=21) *All data are summarized as mean ± standard deviation (SD). BMI, body mass index; C, center; DE, defensive end; DT, defensive tackle; G, guard; NFL, National Football League; OT, offensive tackle; TE, tight end; WR, wide receiver

Characteristics*	Values
Age (y)	27.5 ± 2.9 (range, 23-35)
BMI (kg/m^2^)	32.0 ± 4.2 (range, 26-41)
Seasons Played Before Index Year	4.4 ± 3.0 (range, 1-14)
Games Played During Index Year	5.8 ± 4.0 (range, 1-14)
Draft Position	
Picks 1-16	5 (23.8%)
Picks 17-Undrafted	16 (76.2%)
Injury Timing Per Game	
First Quarter	3 (14.3%)
Second Quarter	8 (38.1%)
Third Quarter	3 (14.3%)
Fourth Quarter	7 (33.3%)
Injury Timing Per Season	
First Quarter (Games One to Four)	9 (42.9%)
Second Quarter (Games Five to Eight)	7 (33.3%)
Third Quarter (Games Nine to Twelve)	3 (14.3%)
Fourth Quarter (Games Thirteen to Sixteen)	2 (9.5%)
Injury Characteristics	
Left Foot	14 (66.7%)
Right Foot	7 (33.3%)
Surgery Performed	18 (85.7%)
Player Position	
Quarterback	3 (14.3%)
Running Back	2 (9.5%)
Receiver (WR, TE)	2 (9.5%)
Offensive Linemen (G, C, OT)	5 (23.8%)
Defensive Linemen (DE, DT)	4 (19.0%)
Linebacker	3 (14.3%)
Corner Back	2 (9.5%)

There was no significant difference in the number of games played one-year pre- and post-index years (median 15.0 (IQR: 12.5 - 16.0) vs. 15.0 (IQR: 12.0 - 16.0); p=0.62). Most players returned to the same team they played for prior to injury (13/16 = 81.3%), and there was no significant difference between any return-to-play statistics between them and those who played for a different team in the season following their injury (Table 2).

**Table 2:** Comparisons of Return-To-Play Statistics Between Players Returning to the Same Team vs. Different Team *All data are summarized as mean ± standard deviation (SD). ^Variables were analyzed using a Welch two-sample t-test. AV, approximate value

Variable*	Returned to the Same Team (n=13)	Returned to a Different Team (n=3)	P-Value^
Snaps Per Game During Index Year	51.7 ± 13.9	54.2 ± 20.4	0.86
Snaps Played Per Game One-Year Post-Index Year	45.6 ± 16.3	47.6 ± 21.1	0.89
Seasons Played Before Index Year	3.4 ± 2.2	5.7 ± 2.1	0.19
Seasons Played Post-Index Year	3.2 ± 2.0	2.3 ± 1.5	0.44
AV One-Year Pre-Index Year	5.2 ± 4.1	8.3 ± 5.1	0.41
AV One-Year Post-Index Year	5.2 ± 3.2	5.0 ± 6.1	0.97

When comparing our injured player's cohort who had return-to-play data (n=16) with the 32 randomly selected control player cohort, there were no significant differences post-injury between the average number of seasons played, AV, and snaps played per game (Table 3).

**Table 3:** Return-To-Play Between Injured Players and Controls *Data summarized as mean ± standard deviation (SD), except for games played (summarized as median (IQR), due to skewness). ^Variables were analyzed using a Welch two-sample t-test, except for games played (analyzed using a Wilcoxon rank sum test due to skewness). AV, approximate value; BMI, body mass index; IQR, interquartile range

Variable*	Cases (n=16)	Control (n=32)	P-Value^
Age (y)	26.8 ± 2.4	26.9 ± 2.6	0.87
BMI (kg/m^2^)	32.2 ± 4.0	33.3 ± 5.0	0.41
Seasons Played Before Index Year	3.8 ± 2.3	4.1 ± 2.2	0.69
AV One-Year Pre-Index Year	5.8 ± 4.3	6.5 ± 3.3	0.58
Snaps Per Game During Index Year	52.2 ± 14.6	48.7 ± 15.0	0.45
Seasons Played Post-Index Year	3.1 ± 1.9	3.7 ± 2.1	0.34
AV One-Year Post-Index Year	5.1 ± 3.6	5.7 ± 3.7	0.64
Snaps Played Per Game Post-Index Year	46.0 ± 16.5	49.0 ± 19.9	0.57
Games Played One-Year Post-Index Year	15.0 (12 – 16)	15.0 (11 – 16)	0.93

## Discussion

Our study analyzed 21 NFL players who sustained a Lisfranc injury during regular season play from 2013 to 2021. It was found that the majority of these injuries (76.2%) occurred during the first eight games of the season (42.9% in the first four games and 33.3% in the second four games). However, the results do not suggest that an increase in snap counts led to an increased incidence of tarsal-metatarsal injury, as players played significantly fewer snaps prior to sustaining their injury compared to their season average. There was also no significant difference found among return-to-play statistics (AV or snaps per game) when comparing the injured cohort to the control cohort. These findings are similar to those noted by McHale et al. who found no statistically significant change in pre- and post-injury athletic performances (offensive power ratings and defensive power ratings) by NFL offensive and defensive players who sustained a Lisfranc injury between 2000 and 2010 [3]. Overall, our study demonstrated that there was not an increase in snap counts contributing to a player’s Lisfranc injury, suggesting that this is more likely an acute event rather than one related to repetitive stress. Offensive and defensive linemen accounted for 42.8% of injuries, which may be due to them having higher BMIs than other positions on average and performing a lot of planting maneuvers that can cause excess load being placed on their feet [21]. It can also be due to them being at an increased risk of opposing players falling or stepping on their feet than other positions. Interestingly, an injury to the left foot was seen twice as frequently as the right, which may be due to the stance and position of their feet, which has been shown to impact injury risk [22].

A search of recent orthopedic literature reveals studies that have attempted to identify the most common points in the season at which NFL players are injured. Baker et al. analyzed the number of NFL injuries across the first four preseason weeks and the first four regular season weeks for the years 2016 and 2018-20 [23]. In 2016, 55% of the documented injuries in weeks one through four occurred in the first two weeks. In 2018, 57% of the documented injuries occurred in the pre-season. In 2019, 58% of the reported injuries occurred in the pre-season. Due to COVID-19, the 2020 pre-season was canceled. However, researchers reported that 51% of the reported regular season injuries between weeks one through four occurred in the first two weeks. These findings suggest a possible positive correlation between the number of NFL injuries and the start of the season. Without proper pre-season training, researchers hypothesize that injuries are more common early in the regular season [23]. Our study builds on this since the majority of Lisfranc injuries occurred during the first eight games of the regular season. Further research would need to be conducted to analyze the efficacy of pre-season training programs in injury prevention.

Acute-chronic workload ratios (AWCRs) have been implemented in orthopedic research as a method to identify the impact of a physical workload with regard to athletic injuries. A 2019 study of English Premier League soccer players found that spikes in ACWRs were associated with a five to seven times greater injury rate [24]. Additionally, it has been found that professional athletes face a greater risk of experiencing a time-loss injury if the ACWR is higher in comparison to a moderate or a lower ACWR [25]. While we were not able to calculate an ACWR with the available data, our study points toward a more acute cause of Lisfranc injuries in the NFL and supports future research that could be directed toward the computation of ACWRs to identify a potential activity threshold for these injuries. 

While this study did not find a correlation between an increased number of snap counts and the frequency of Lisfranc injuries in the NFL, future research could be directed toward investigating potential practices that can lead to the prevention of such injuries. Our study suggests an acute cause for injury as most injuries occurred in the first half of the NFL regular season. This suggests a possible need for further exploration of the environment and equipment that NFL players compete in and utilize. Future research could help identify potential elements of an NFL game that make a player more susceptible to a Lisfranc injury. For instance, the protective or injurious features of particular footwear or the composition of the NFL football field (artificial turf in comparison to grass) could be investigated.

This study was not without limitations. Players with Lisfranc injuries were identified through public resources, and as a result, anyone who was not reported in public databases would not be included in this study. Also, surgical techniques, physical examinations, and rehabilitation protocols could not be assessed based on the inability to acquire individual medical records. Variations of these factors could lead to differences in a player’s ability to return to sport and perform at a level they were previously.

## Conclusions

Our study analyzed 21 NFL players who sustained a Lisfranc injury during the 2013 and 2021 NFL seasons. Increased snap counts were not seen prior to a Lisfranc injury in the NFL. Offensive and defensive linemen accounted for the greatest percentage of injuries at 42.8%. Finally, it was found that the majority of these injuries (76.2%) occurred during the first eight games of the season (42.9% in the first four games and 33.3% in the second four games).
